# An examination of the effectiveness of health warning labels on smokeless tobacco products in four states in India: findings from the TCP India cohort survey

**DOI:** 10.1186/s12889-016-3899-7

**Published:** 2016-12-13

**Authors:** Shannon Gravely, Geoffrey T. Fong, Pete Driezen, Steve Xu, Anne C. K. Quah, Genevieve Sansone, Prakash C. Gupta, Mangesh S. Pednekar

**Affiliations:** 1 Department of Psychology, University of Waterloo, 200 University Avenue West, Waterloo, ON N2L 3G1 Canada; 2 Institute for Cancer Research, Toronto, Canada; 3 School of Public Health and Health Systems, University of Waterloo, Waterloo, ON Canada; 4 Healis-Sekhsaria Institute for Public Health, Navi Mumbai, India

**Keywords:** Tobacco control, Health policy, Health warning labels, Smokeless tobacco

## Abstract

**Background:**

In 2009, after many delays and changes, India introduced a single pictorial health warning label (HWL) on smokeless tobacco (SLT) packing—a symbolic image of a scorpion covering 40% of the front surface. In 2011, the scorpion was replaced with 4 graphic images. This paper tested the effectiveness of SLT HWLs in India and whether the 2011 change from symbolic to graphic images increased their effectiveness.

**Methods:**

Data were from a cohort of 4733 adult SLT users (age15+) of the Tobacco Control Project (TCP) India Survey from 4 states. The surveys included key indicators of health warning effectiveness, including warning salience, and cognitive, emotional, and behavioral responses to the warnings.

**Results:**

The HWL change from symbolic to graphic did not result in significant increases on any of the HWL outcome indicators. A substantial minority of SLT users were unaware that SLT packages contained HWLs (27% at both waves). Noticing the warnings was also remarkably low at both waves (W1 = 34.3%, W2 = 28.1%). These effects carried over to the cognitive and behavioural measures, where among those who noticed HWLs, about one-third reported forgoing SLT at least once because of the HWLs, and fewer than 20% reported that HWLs made them think about SLT risks or about quitting SLT. Even fewer reported avoiding HWLs (8.1 to 11.6%). Among those who quit using SLT by post-policy, awareness that SLT packaging contained HWLs was significantly greater at post-policy (86.8%) compared to pre-policy (77.8%, *p* = 0.02). Quitters were also significantly more aware of the post-policy HWLs compared to those who continued to use SLT (*p* < 0.001).

**Conclusions:**

Health warnings on SLT packages in India are low in effectiveness, and the change from the symbolic warning (pre-policy) to graphic HWLs (post-policy) did not lead to significant increases of effectiveness on any of the HWL indicators among those who continued to use SLT products, thus suggesting that changing an image alone is not enough to have an impact. There is a critical need to implement SLT HWLs in India that are more salient (large in size and on the front and back of the package) and impactful, which following from studies of HWLs on cigarette packaging, would have strong potential to increase awareness of the harms of SLT and to motivate quitting.

**Electronic supplementary material:**

The online version of this article (doi:10.1186/s12889-016-3899-7) contains supplementary material, which is available to authorized users.

## Background

Over the last decade ―since the WHO Framework Convention on Tobacco Control (FCTC) came into force in 2005― there has been strong progress in tobacco control in many countries around the world. These efforts however have been largely focused on smoked tobacco (mainly manufactured cigarettes) with limited attention paid to other harmful and prevalent tobacco products, such as smokeless tobacco (SLT).

Currently, more than 300 million adults in 70 countries across all WHO regions use SLT, of which 250 million (90% of global SLT users) are in the 11 countries of the WHO’s South East Asian Region (SEAR) [1]. Notably, rural users in India and Bangladesh make up 80% of the total SLT users in the world [2]. Moreover, studies have shown that SLT use is highest among SEAR’s illiterate and low socioeconomic populations [1].

SLT products represent a significant risk to human health [3–6]. There is considerable diversity of SLT products, but those commonly used in the SEAR, and notably in India, include highly toxic forms, with very high levels of harmful constituents such as nitrosamines and heavy metals [2, 7, 8]. SLT products in SEAR have been shown to cause a broad range of diseases and adverse health effects such as various types of cancers, cardiovascular disease, and adverse pregnancy outcomes [1, 2, 5, 6, 9–11]. The latter is particularly striking, as SLT use is highly prevalent among reproductive age females in SEAR [11, 12].

Despite the considerable evidence base linking SLT use with adverse health outcomes, knowledge of the health effects of SLT remains low [6, 13, 14]. Added to this, there are many misconceptions about SLT, including the belief among many that SLT can be used for health purposes, such as cleansing their teeth and to relieve stress and other health ailments. These misconceptions have likely contributed to low rates of quitting among SLT users in many of these countries [1, 15, 16].

Due to a lack of public awareness and incomplete knowledge about the harmful effects of SLT, there are significant challenges towards effective tobacco control related to SLT use. One of the most effective public health measures to inform the people about the harms of tobacco products is to implement large pictorial health warning labels (HWLs) [17]. Overall, many collective elements, such as the size, position, content, and design of these messages, influence HWL effectiveness [18–20]; however, although there has been a great deal of global research on the effectiveness of tobacco HWLs on manufactured cigarette packages, there is little evidence about the impact of these warnings on SLT packaging [2, 21]. This is particularly true in the SEAR, but most notably in India where SLT use is highly prevalent, and where it exceeds smoked tobacco among both men and women [2].

### Smokeless tobacco and health warning labels in India

About 275 million people currently use tobacco in India. Among all tobacco products, SLT is the predominant form used by men (32.9%), women (18.4%), and youth (9.0%); it exceeds the prevalence of cigarette smoking [22] and that of other smoked products (e.g., bidis) [16]. Men and women also differ in the types of SLT products that they use, and thus are exposed to many different forms of product packaging, which would likely further reduce the impact of HWL by gender [23].

Although India’s *2003 Cigarette and Other Tobacco Products Act* called for pictorial warnings on both smoked and smokeless tobacco product packaging, tobacco industry influence led to years of delays and dilutions [22, 24, 25]. When pictorial HWLs were finally introduced in 2009, they were weakened so that they did not meet the WHO FCTC Article 11 Guidelines [26]. From May 2009 to November 2011, SLT packages included a HWL with one symbolic image (that of a scorpion, which is unrelated to cancer) on 40% of the front of the package. In December 2011, the scorpion image was replaced by 4 graphic images of cancer of the mouth, jaw, or neck [27]. In 2013, 3 new graphic HWLs were implemented. All SLT warnings since 2011 were accompanied by the text “TOBACCO KILLS”; however, the size (40%), location (on the front only), and the lack of rotation remained [22, 25]. The tobacco industry could also freely choose only one of the available warnings [28], http://www.tobaccolabels.ca/countries/india/.  

Cross-sectional and qualitative studies show that the 2009 scorpion HWL was poorly understood [29–31], but to our knowledge, there are no longitudinal population studies that have examined the effectiveness of the change from the 2009 scorpion warning to the graphic 2011 HWLs. Therefore the objectives of this paper were to test: (1) the effectiveness of the 2009 and 2011 SLT HWLs in India; and (2) whether the 2011 change from symbolic to graphic images increased effectiveness among validated HWL indicators and intentions to quit SLT use. We conducted this evaluation with a longitudinal cohort design, which confers considerable advantages in policy evaluation [32].

## Methods

### Sample design and procedure

This study is part of the larger TCP India Survey, which is a prospective cohort study of adult (aged ≥15 years) tobacco users and non-users from 4 states in India: Bihar, West Bengal, Madhya Pradesh, and Maharashtra. In each state, residents of the following urban cities and their surrounding rural districts were surveyed: Patna (Bihar), Kolkata (West Bengal), Indore (Madhya Pradesh), and Mumbai (Maharashtra). Within each state, one major city was selected to represent an urban area and the surrounding area within a 50 km diameter outside of the major city was selected to represent a rural area.

At Wave 1, the TCP India Survey followed a stratified multistage cluster sampling design. In order to adjust for potential disproportionate selection of adult tobacco users and non-users in subgroups, enumeration and survey weights were calculated for each enumerated household and survey respondent. Means and proportions reported here were estimated using longitudinal sampling weights that adjust for respondent attrition and are interpreted as the number of people in the population that a respondent represents. Wave 1 of the TCP India Survey was conducted between August 2010 and October 2011, and Wave 2 was conducted between October 2012 and September 2013. Further details about household enumeration in the four states, the study sampling design, the construction of sampling weights, the selection criteria for survey respondents in each household, and the response rates are provided in the TCP India Technical Reports [33].

Fieldwork was conducted by the Healis-Sekhsaria Institute for Public Health in Maharashtra; the School of Preventative Oncology in Bihar; the Madhya Pradesh Voluntary Health Association (MPVHA) in Madhya Pradesh; and the Cancer Foundation of India in West Bengal. Healis led the conduct of the survey in all four states. For the Wave 1 and 2 Surveys, the protocol and questionnaires were first developed in English and then translated into the dominant languages spoken in each state. Respondents answered the survey in their preferred language: English or in Hindi in Bihar and Madhya Pradesh, Marathi in Maharashtra, and Bengali in West Bengal. The average length of the survey interview was 96 min at Wave 1 and 101 min at Wave 2. At the end of the interview, respondents were debriefed, remunerated, and thanked for their time. A token of appreciation was presented to each respondent which was a gift equivalent to $3.00 USD (further details are described in the TCP technical reports) [33]. Ethics clearance was granted by the University of Waterloo, Office of Research Ethics and the Healis-Sekhsaria Institute for Public Health Institutional Review Board.

### Study sample

Data for this study were drawn from the larger TCP India Survey and included current SLT users that participated at both Wave 1 and Wave 2. A SLT user was defined as *use of any smokeless tobacco products at least once a month.* The demographic and SLT characteristics of the sample for Waves 1 and 2 are shown in Table 1.

**Table 1:** Respondent’s baseline demographic characteristics and smokeless tobacco use behaviours

Characteristic, *n* (%)	Maharashtra	Bihar	Madhya Pradesh	West Bengal	Total Sample*	*P*-Value
	*n* = 1101 (23.3%)	*n* = 1551 (32.8%)	*n* = 1168 (24.7%)	*n* = 931 (19.3%)	*N* = 4733	
Sex, *n* (%)						
Male	514 (46.7)	887 (57.2)	839 (71.8)	426 (46.7)	2666 (56.3)	<0.001
Female	587 (53.3)	664 (42.8)	329 (28.2)	487 (53.3)	2067 (43.7)	
Age, *n* (%)						
15–17	4 (0.4)	98 (6.3)	22 (1.9)	9 (1.0)	133 (2.8)	<0.001
18–24	46 (4.2)	258 (16.6)	140 (12.0)	87 (9.5)	531 (11.2)	
25–39	351 (31.9)	536 (34.6)	379 (32.4)	281 (30.8)	1547 (32.7)	
40–54	382 (34.7)	388 (25.0)	335 (28.7)	305 (33.4)	1410 (29.8)	
55+	318 (28.9)	271 (17.5)	292 (25.0)	231 (25.3)	1112 (23.5)	
Marital status, *n* (%)						
Married	831 (75.5)	1111 (71.6)	832 (71.5)	663 (72.9)	3437 (72.7)	<0.001
Single	74 (6.7)	344 (22.2)	179 (15.4)	103 (11.3)	558 (12.4)	
Other	196 (17.8)	96 (6.2)	152 (13.1)	144 (15.8)	700 (14.8)	
Education level, *n* (%)						
Low	665 (60.5)	845 (54.5)	739 (63.3)	718 (79.0)	2967 (62.8)	<0.001
Moderate	408 (37.1)	446 (28.8)	336 (28.8)	140 (15.4)	1330 (28.1)	
High	27 (2.5)	260 (16.8)	92 (7.9)	51 (5.6)	430 (9.1)	
Income level, *n* (%)						
Low	112 (10.2)	454 (29.3)	348 (29.8)	420 (46.0)	1334 (28.2)	<.001
Moderate	777 (70.6)	834 (53.8)	687 (58.8)	381 (41.7)	2679 (56.6)	
High	172 (15.6)	236 (15.2)	89 (7.6)	92 (10.1)	589 (12.4)	
Not stated	40 (3.6)	27 (1.7)	44 (3.8)	20 (2.2)	131 (2.8)	
District type, *n* (%)						
Urban	743 (67.5)	1127 (72.7)	892 (76.4)	691 (75.7)	3453 (73.0)	0.75
Rural	358 (32.5)	424 (27.3)	276 (23.6)	222 (24.3)	1280 (27.0)	
Number of SLT products used, *n* (%)						
1	839 (76.2)	1224 (78.9)	773 (66.2)	673 (73.7)	3509 (74.2)	0.039
2	210 (19.1)	254 (16.4)	302 (25.9)	193 (21.1)	959 (20.3)	
3	52 (4.7)	73 (4.7)	92 (7.9)	47 (5.1)	264 (5.6)	
Use frequency, *n* (%)						
Less than daily	7 (0.6)	59 (3.8)	7 (0.6)	20 (2.2)	93 (2.0)	<0.001
Almost daily	103 (9.4)	565 (36.4)	200 (17.1)	146 (16.0)	1014 (21.4)	
More than once/day	991 (90.0)	927 (59.8)	960 (82.3)	745 (81.8)	3623 (76.6)	
SLT quit attempt (ever), *n* (%)						
Yes	302 (27.5)	395 (25.6)	304 (26.2)	176 (19.3)	1177 (25.0)	0.49
No	798 (72.5)	114 (74.4)	858 (73.8)	734 (80.7)	3538 (75.0)	
Daily SLT consumption (number of times per day)						
≤ 10	1407 (91.5)	731 (81.0)	915 (79.4)	1008 (91.7)	4061 (86.6)	<0.001
11–20	116 (7.5)	138 (15.3)	205 (17.8)	74 (6.7)	533 (11.4)	
21–30	10 (0.7)	24 (2.7)	20 (1.7)	12 (1.1)	66 (1.4)	
31+	4 (0.3)	9 (1.0)	13 (1.1)	5 (0.5)	31 (0.7)	
Time to first use after waking (min)						
> 60	573 (37.1)	338 (37.4)	125 (10.7)	291 (26.5)	1327 (28.1)	< 0.001
31–60	344 (22.3)	121 (13.4)	291 (24.9)	88 (8.0)	844 (17.9)	
6–30	416 (26.9)	252 (27.9)	572 (49.0)	353 (32.1)	1593 (33.8)	
≤ 5	211 (13.7)	193 (21.3)	180 (15.4)	367 (33.4)	951 (20.2)	
SLT quit attempt (in the last year, *n* (%)						
Yes	116 (10.5)	296 (19.2)	285 (24.6)	117 (12.9)	814 (17.3)	
No	984 (89.5)	1243 (80.8)	875 (75.4)	790 (87.1)	3892 (82.7)	0.003

Some characteristics have missing values if they were not reported at time of entry into the study (percentages take into account missing data). Results are unweighted but the survey design was accounted for in the analysis. All tests are the Rao–Scott *χ*2 test unless otherwise indicated

### Measures

#### Smokeless tobacco users

All respondents were asked the following question at both Waves 1 and 2: ‘*Do you currently use any of the following smokeless tobacco products at least once a month’* (yes/no/don’t know). Those who responded *‘yes’* at both waves were included as a *"SLT user"* for the current study. Respondents also identified what type of SLT products that they use. SLT products included (1) those for chewing or holding in the mouth: plain chewing tobacco, slaked lime mixed with tobacco, known as khaini; scented chewing tobacco, known as zarda; (2) areca nut and tobacco mixtures for chewing: gutka, industrially made crushed areca nut with tobacco; betel quid, which is areca nut, tobacco and condiments wrapped in a fresh betel leaf, prepared by vendors or at home; and (3) products used as dentifrices or for application to teeth and gums: dry snuff (also called bajjar or tapkheer), gudhaku (a paste of tobacco and molasses) tobacco toothpaste, pyrolized products (gul and mishri), Lal dantmanjan (red toothpowder).

#### Demographics and tobacco related variables

Sociodemographic characteristics were assessed with standard questions on sex, age, marital status, highest educational attainment, and monthly household income (equivalent to US dollar, 1 Indian Rupee (INR) decreased from 0.23 to 0.15 US dollars during the study period). Income and education were categorized as low, moderate, and high. Monthly household income was categorized as “low” (5000 INR or less), “moderate” (5001–15,000 INR), and “high” (15,001 INR or higher). Those who were illiterate, literate with no formal education, completed up to primary school, or middle school were categorized as “low education”. Those who completed secondary school were categorized as “moderate education” and those who completed graduate, post-graduate or professional degree or above were categorized as “high education”.

Tobacco-relevant variables consisted of: SLT use frequency (*On average, how often do you use this product (your most frequent smokeless product: Less than once a week, Once a week, Twice a week, 3-5 times a week, Every day or almost every day, More than once a day*), dependence *(analogous to the heaviness of smoking index, in this case, the sum of the categorical measures of the daily amount of smokeless used (0 = ≤ 10/day, 1 = 11–20/day, 2 = 21–30/day and 3 = 31+/day) and the time to first use (0 = > 60 min after waking, 1 = 31–60 min after waking, 2 = 6 to 30 min after waking and 3 = within 5 min after waking). The dependence scale therefore took on values from 0 to 6),* previous cessation attempts *(Have you ever made a serious attempt to stop using all smokeless tobacco products* and *Have you made a serious attempt to stop using all smokeless tobacco products in the last year (Yes/no),* and intentions to quit *(Are you planning to quit using smokeless tobacco: Within the next month; Within the next 6 months; Sometime in the future beyond 6 months; Not planning to quit)*. All questions included an option of “refused” or “don’t know”.

#### Health warning effectiveness measures

Conceptual work and empirical studies have identified key indicators of HWL effectiveness, which have been employed in a wide range of studies across different countries [19, 20, 32]. These key indicators were:

1. *Awareness* of HWLs on SLT packaging was assessed by asking the question:
  “Thinking now about the packages for smokeless tobacco products (paste, sachets, packs, tins, bottles), as far as you know, do any smokeless tobacco packages in India have warning labels?” The response option were: (yes, no, don’t know).
2. HWL *salience (noticing* and *reading* the warnings closely) was assessed by two questions:
  i. “In the last month, how often have you noticed the health warnings on smokeless tobacco packages?” and
  ii. “In the last month, how often have you read or looked closely at the health warnings on smokeless tobacco packages?”
  The response options for both were: “Never,” “Once in a while,” “Often,” “Very often, and whenever I use smokeless tobacco.” These responses were dichotomized into “at least once in a while” vs “Never”.
3. *Cognitive* reactions to the HWLs were: (i) *thoughts about the harms of SLT;* and (ii) *thoughts about quitting* and were assessed with the following two questions:
  i. “To what extent, if at all, do the warning labels on smokeless tobacco packages make you more likely to think about the health risks (health danger) of using it?” and
  ii. “To what extent, if at all, do the warning labels on smokeless tobacco packages make you more likely to quit using it?”
  The response options were: “Not at all,” “A little,” and “A lot” and dichotomized into “at least a little” vs “not at all”.
4. *Behavioral* reactions to the HWLs were *forgoing SLT* use and *avoidance of warnings* and were assessed by asking:
  i. “In the last month, have the warning labels stopped you from using smokeless tobacco when you were about to use it?” Response options were: “Never,” “Once,” “A couple of times,” “Once in a while”, and “Many times”. This was dichotomized into “at least once” vs “never”.
  ii. “In the last month, have you made any effort to avoid looking at or thinking about the smokeless tobacco warning labels -- such as covering them up, keeping them out of sight, avoiding certain warnings, or any other means?” (Yes/No).

### Statistical analyses

Initial unweighted descriptive statistics were computed for demographic variables and tobacco use characteristics (see Table 1), and weighted estimates were computed for each outcome measure (HWL outcomes and intentions to quit) at each wave.

Binary logistic regression models using generalized estimated equations (GEE) were used to: (1) estimate a weighted, adjusted percentage for each outcome measure pre- and post-policy; and (2) test the difference in pre- and post-policy estimates. All models employed an exchangeable working correlation structure.

The analyses were conducted using SUDAAN (Version 11.0.1), which took into account the multistage sampling design and the longitudinal data. All analyses adjusted for survey wave, state, urban/rural area, sex, age group, marital status, income, education, attempt to quit SLT in the past year, intention to quit SLT, and SLT dependence. The analyses included SLT users that were present both at pre and post-policy and that used SLT products only (to reduce bias from dual use of smoked and smokeless products). People who no longer used SLT at the time of the Wave 1 or Wave 2 survey were excluded from the main analyses. Unless otherwise stated, all results were weighted.

Three sub-analyses were conducted to test if: (1) there were differences in types of SLT products used by gender; (2) HWL effectiveness among respondents that reported use of a SLT product with mandated HWLs only (gutkha, chewing tobacco, zadra, snuff and khaini) increased after the 2011 policy change; and (3) HWL awareness and noticeability differed pre-and post-policy among SLT users at pre-policy but had quit by post-policy (as the cessation survey only included these 2 indicator outcomes), and if quitters were more or less likely to be aware of, or notice the labels by post-policy compared to the respondents that continued to use SLT.

## Results

### Pre- and post-policy survey sample differences

**Fig. 1:**
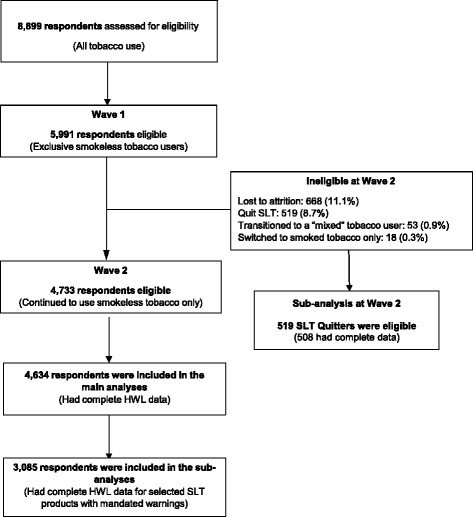
Study Flow Diagram

The study flow diagram is presented in Fig. 1.

At pre-policy, 5991 respondents reported exclusive use of SLT. At post-policy 668 were lost to attrition and 590 were excluded, leaving 4733 for analyses. Unweighted analyses showed that respondents lost to attrition were more likely to be from Maharashtra (*p* < 0.001), from an urban area (*p* < 0.001), younger (*p* < 0.01), male (*p* < 0.001), did not make an attempt to quit SLT in the last year (*p* < 0.01), and had lower education (*p* < 0.001).

### Respondent characteristics

Overall, 56.3% of the sample was male, with an average age of 41.8 years ± 15.8. The majority of the respondents were married (72.7%), of moderate income (56.6%), with a low education (62.8%), lived in an urban area (73.0), and 24.2% were illiterate. Seventy-four percent of respondents used one type of SLT product, 76.6% used SLT more than once a day, and 75% had never tried to quit using SLT.

**Fig. 2:**
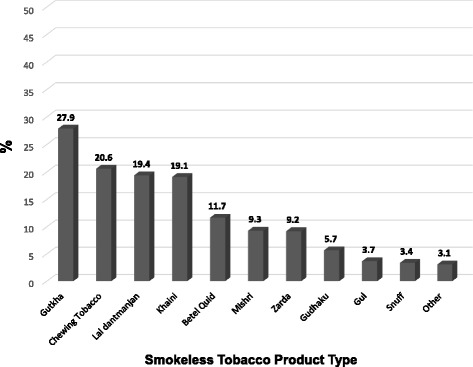
Types of smokeless tobacco products used among India cohort respondents from all four States. Note that SLT groups are not mutually exclusive (Respondents could have reported more than 1 type)

Figure 2 shows the self-reported use of various SLT products among all respondents, and Additional file 1: Table S1 shows SLT type of products used by respondents using only one product.

When SLT product use was examined by gender, men were significantly more likely to use SLT products with mandated HWLs: gutkha (40.2% vs. 11.9%, *p* < 0.001), zarda (10.3% vs. 7.8%, *p* = 0.009), chewing tobacco (26.0% vs. 13.6%, *p* < 0.001), and khaini (32.3% vs. 2.2%, *p* < 0.001). Women were more likely to use products without mandated HWLs: mishri (17.9%, vs. 2.6% *p* < 0.0010, gudhaku (11.0% vs. 1.6%, *p* < 0.001), Gul (5.6% vs. 2.1%, *p* < 0.001), and lal dantmanjan (31.5% vs. 10.1%, *p* < 0.001), although women (6.0%) were more likely to use snuff compared to men (1.4%, *p* < 0.001). There were no sex differences in betel quid use.

### SLT User’s responses to the HWL changes

Table 2 presents the results from the GEE adjusted analysis for the 7 HWL indicators, and for quit intentions.

**Table 2:** GEE analysis examining differences in health warning label impact on smokeless tobacco user’s salience, perceptions, behaviour and intensions to quit smokeless between pre- and post-policy periods (Waves 1 and 2)

	Wave			Wave						
	1			2			Difference between Waves			
Outcome	%	(95%	CI)	%	(95%	CI)	Diff	SE Diff	Test	*p* value
All respondents (*N* = 4634)										
Aware that SLT packages contain HWLs (yes)	72.7	67.1	77.7	73.0	67.3	78.1	0.3	2.9	0.1	0.92
Noticed HWLs at least once in a while (yes)	34.3	28.5	40.6	28.1	21.8	35.4	−6.2	4.0	−1.5	0.13
Among respondents that noticed HWLs (*n* = 2154)										
Read HWLs at least once in a while (yes)	49.4	42.0	56.9	50.1	40.4	59.9	0.7	6.4	0.1	0.92
HWLs made you think about risks of SLT at least a little (yes)	15.0	11.9	18.8	17.5	12.1	24.6	2.5	3.1	0.8	0.42
HWLs made you think about quitting SLT at least a little (yes)	16.8	13.0	21.4	19.3	13.6	26.6	2.5	3.1	0.8	0.422
Avoided looking at HWLs (yes)	8.1	5.5	11.8	11.6	7.8	17.0	3.6	2.0	1.7	0.09
Gave up SLT at least once because of HWLs (yes)	31.3	24.3	39.3	36.7	27.2	47.5	5.4	4.9	1.1	0.27
Any intentions to quit SLT (yes)	19.8	14.6	26.4	20.5	15.2	27.0	0.6	4.5	0.1	0.89

All estimates are weighted; *CI* Confidence interval, *SLT* Smokeless tobacco, *HWL* Health warning label, *SE* Standard error, *p* probability (based on *P* < 0.05); The data were adjusted with the following covariates: State, sex, urban/rural, age, marital status, income, education, quit attempt in last year, intentions to quit, wave and SLT dependence

Prior to and after controlling for covariates, the change from symbolic (pre-policy) to graphic (post-policy) HWLs did not lead to significant increases for any of the HWL indicators. There were no differences between pre- (72.7%) and post-policy (73.0%) user’s awareness that SLT packages contained a HWL. Respondents from Bihar and West Bengal, women, older age (those aged 55+), those of lower and moderate education, and SLT users with no intention to quit were significantly less likely than others to respond “yes” to awareness of the HWLs. Additionally, there were no differences between pre- (34.3%), and post-policy (28.1%) for noticing the HWLs (*p* = 0.13).

Among respondents who were aware of HWLs, half of the sample reported reading the HWLs at least once in a while (pre-policy = 49.4%, post-policy = 50.1%, *p* = 0.92), 15.0% of users at pre-policy and 17.5% at post-policy reported that HWLs made them think about SLT risks (*p* = 0.42), 16.8% at pre-policy and 19.3% at post-policy reported that HWLs made them think about quitting SLT (*p* = 0.42), and 31.3% at pre-policy and 36.7% at post-policy reported giving up SLT at least once because of the HWL (*p* = 0.27). There were low levels of HWL avoidance: 8.1% of users at pre-policy and 11.6% at post-policy (*p* = 0.09). Additionally, there were no changes between pre- (19.8%) and post-policy (20.5%) towards greater intentions to quit SLT due to the HWLs (*p* = 0.89). Similarly, when users of SLT products with mandated HWLs only were selected for analysis (*n* = 3085), there remained no significant changes for any of the HWL indicators or intentions to quit (see Table 3).

**Table 3:** GEE analysis examining differences in health warning labels on smokeless tobacco user’s salience, perceptions, behaviour and intensions to quit smokeless between pre- and post-policy periods (Waves 1 and 2) among those that used SLT with HWL mandated packaging, *n* = 3085

	Wave			Wave					
	1			2			Difference between Waves		
Outcome	%	(95%	CI)	%	(95%	CI)	Diff	Test	*p* value
Aware that SLT packages contain HWLs (yes)	78.7	72.9	83.6	80.4	75.4	84.6	1.7	0.6	0.52
Noticed HWLs at least once in a while (yes)	38.4	31.9	45.3	31.8	25.1	39.4	−6.5	−1.6	0.11
Read HWLs at least once in a while (yes)	20.8	16.4	26.1	17.5	12.8	23.5	−3.3	−0.9	0.35
HWLs made you think about risks of SLT at least a little (yes)	9.6	7.3	12.4	10.0	6.6	14.8	0.5	0.2	0.86
HWLs made you think about quitting SLT at least a little (yes)	9.5	6.8	13.0	11.3	7.7	16.2	1.8	0.7	0.52
Avoided looking at HWLs (yes)	4.5	3.1	6.5	5.4	3.7	8.0	0.9	0.8	0.45
Gave up SLT at least once because of HWLs (yes)	17.6	13.9	22.1	22.0	16.2	29.1	4.4	1.3	0.19
Any intentions to quit SLT (yes)	14.2	10.4	19.1	12.4	9.2	16.6	−1.8	−0.7	0.511

All estimates are weighted; *CI* Confidence interval, *SLT* Smokeless tobacco, *HWL* Health warning label, *SE* Standard error, *p* probability (based on *P* < 0.05); The data were adjusted with the following covariates: State, sex, urban/rural, age, marital status, income, education, quit attempt in last year, intentions to quit, wave and SLT dependence

### Quitters’ responses to the HWL changes

There were 519 (8.7%) respondents that self-reported having quit SLT use by post-policy (508 had complete data for analyses). In an adjusted GEE analysis, SLT quitters were added to the main sample so that all respondents that completed a post-policy survey were included (*N* = 5142). Overall, the estimates were similar to the results among the continued users (see Additional file 2: Table S2).

Next, an adjusted GEE that included SLT quitters only, showed that awareness that SLT packaging contained HWLs was significantly greater at post-policy (86.8%) compared to pre-policy (77.8%, *p* = 0.02). Quitters were also significantly more aware of the post-policy HWLs compared to those who continued to use SLT (*p* < 0.001). Noticing the labels at least once in a while between pre- and post-policy was not significant among the SLT quitters, but there was a statistical trend towards a decrease in noticing the HWLs on SLT packaging at post-policy (*p* = 0.09).

## Discussion

Implementing HWLs that meet the FCTC Article 11 Guidelines is an effective strategy for enhancing their perceptual, cognitive, and behavioural impact on tobacco users. However unlike the strong evidence for the effectiveness of HWLs on smoked tobacco packaging [17, 19, 20], there is little evidence about the effectiveness of HWLs on SLT, and whether enhanced warnings on SLT packaging would impact emotional, cognitive, and behaviour changes among users. This study tested both of these objectives. The results of this first longitudinal study of HWLs on India’s SLT products showed that the effectiveness was very low at both pre- and post-policy, and the change from symbolic to graphic images did not increase any of the HWL indicators or intentions to quit SLT, neither among the entire cohort of SLT users nor among those using SLT products with mandated HWLs. Interestingly however, those that quit SLT use were significantly more aware that SLT packaging contained the HWLs at post-policy compared to the pre-policy measure (and quitters were also significantly more aware of HWLs compared to those that continued to use SLT), thus the new graphic HWLs may have impacted those who quit SLT differently that those who continued use. The trend in the decrease of noticing the HWLs among SLT quitters could be explained by the fact that they were no longer regularly exposed to SLT packaging.

Among continued SLT users, the low level of effectiveness of the HWLs was evident across the indicators: from awareness and salience to the cognitive and behavioural reactions. Indeed, a substantial minority were not even aware that there were HWLs on SLT products (27% reported that they were not aware of the 2009 scorpion image or the 2011 graphic images), which is surprising considering that 90% of the users reported using SLT more than once a day, thus they should have been frequently exposed to the HWLs on SLT packaging. Even when the analyses only included users of SLT products that required mandated HWLs, 20% reported that they were unaware that the package contained health warnings at both pre-and post-policy. This surprising level of unawareness is similar to that found in smaller cross-sectional studies, including 27.5% in Karnataka [31], 20% in the Kumaon Hills of India [34], and 31.2% in Mumbai [30].

**Fig. 3:**
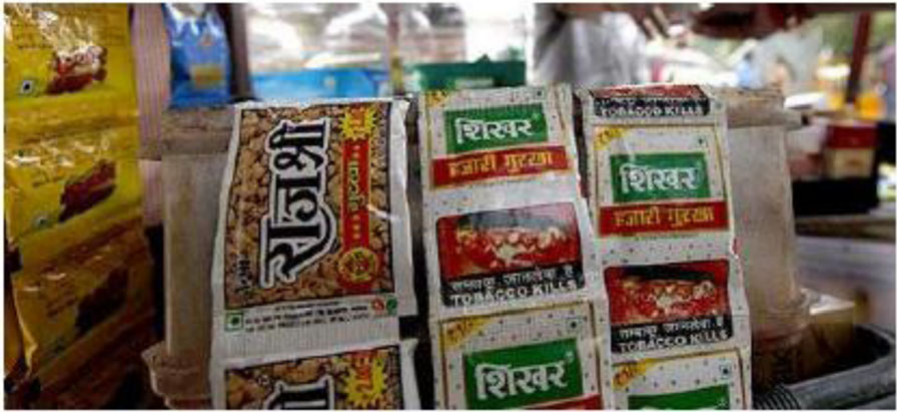
Example of smokeless tobacco packaging in India f4:2 (December 2011 – April 2016)

The results from this study confirm those of other studies in India in which HWLs have low levels of noticing and effectiveness [14, 30, 31, 34]. There are a number of possible reasons for this low level of effectiveness. For example, SLT packages tend to be considerably smaller in size compared to smoked tobacco products such as cigarettes. As a result, the overall salience will be diminished because of the actual size of the warnings. This is particularly true for single-use packages, which are common. Added to the reduced size, a large proportion of users are poorly educated, which would reduce the comprehension of the warnings. Additionally, there is wide variety and diversity of package design used by SLT manufacturers [2], which is a reflection of their use of packaging as a marketing venue to reinforce brand imagery, to minimize perceptions of risk, and to suggest incorrectly that some types of products are less harmful than others. SLT packaging designs can effectively counteract warning content through creative techniques that can undermine the salience and impact of the warnings (see Fig. 3). Because SLT packages are exceptionally vibrant (e.g., multicolored with elaborate graphic designs), HWLs have to compete for attention. Therefore research is needed to examine the HWL design that will have the greatest impact and noticeability on SLT users in India (e.g., they should be much bigger and have contrasting colours to the other packaging design elements) Other reasons for low salience include the fact that some products are made at home, some users may only use SLT in the form of loose tobacco purchased from local farmers or producers (even locally produced and marketed tobacco products with packaging often fail to display HWLs), and many forms of SLT are prohibited from containing tobacco, but often do, thus they do not require a mandated warning (e.g., lal dantmanjan) [35, 36].

The weakness of the SLT warnings was also found in the cognitive and behavioral HWL indicators. For example, 10% of SLT users with mandated HWL packaging reported that the warnings had generated thoughts of quitting, or about the risks of SLT (so this leaves 90% of users not considering the health risks or quitting SLT use). Furthermore, only 20% at both pre-and post-policy had intentions to quit SLT use. Similarly, the study by Mutti et al. [37] reported that Indian SLT users intending to quit SLT rated warnings as more effective than those without any quit intentions [37]. ITC findings demonstrate that India’s HWLs on smoked tobacco products have the lowest level of effectiveness of all 20+ countries including other LMICs [38]. Given that there exist linkages between the effectiveness of health warnings and subsequent intentions to quit [39, 40], and that quit intentions are a strong predictor of future quit attempts [41–43], it is clear that the low effectiveness of health warnings on tobacco products in India represents a considerable lost opportunity for reducing both consumption and prevalence of tobacco use.

The results from this study highlight the critical need to improve the salience and other downstream effects of HWLs on SLT products. To date, the progress of India’s HWLs on both smoked and SLT has reflected a tangled exchange between the tobacco industry and the Indian government [22]. Research evidence on whether the effectiveness of warnings—on both smokeless and smoked tobacco products—will continue to be an important component of efforts to increase the impact of policies in this domain. Notably however, India’s government has implemented new mandated warnings that are among the strongest in the world (as of April 1, 2016). These new labels now cover 85% of the front and back of the principal surfaces of the packaging [44]. The industry however has attempted to fight back by halting tobacco production. The industry is claiming that they paused manufacturing because of confusion over the new HWL requirements, but antismoking activities claim that this was in fact an attempt to put pressure on regulators through means of economic impact (e.g., through losses in employment of factory workers and farmers and tax revenues) [45].

The findings in this study should be interpreted with caution, mainly due to measurement and generalizability. With regard to measurement, we did not ask about a number of variables that might have contributed to the low awareness of the HWLs, including whether their SLT came in a package or container, purchasing of illicit products, or if their SLT product was home-made. Moreover, the TCP Survey was conducted in 4 states in India, and so generalization to India as a whole is not possible. Additionally, the post-policy survey for SLT quitters did not include a question that directly asked if they *quit using SLT because of the HWLs*, therefore no conclusions can be made about the impact of the warnings on their decision to quit.

Although there are some study limitations, this is the first population longitudinal cohort study to examine the impact of HWLs on SLT packages in India. Moreover, this study included a large number of participants, particularly women, rural inhabitants, and low education people where SLT is most prevalent.

## Conclusions

Health warnings on SLT packages in India are low in effectiveness, and the change from the symbolic warning (pre-policy) to graphic HWLs (post-policy) did not lead to significant increases of effectiveness on any of the HWL indicators among those who continued to use SLT products, thus suggesting that changing an image alone is not enough to have an impact. There is a critical need to implement SLT HWLs in India that are more salient (large in size and on the front and back of the package) and impactful, which following from studies of HWLs on cigarette packaging, would have strong potential to increase awareness of the harms of SLT and to motivate quitting.

## Additional files

Additional file 1: Table S1. Type of products used by SLT respondents by state among those who reported using only one product. (DOC 50 kb)
Additional file 2: Table S2. GEE analysis examining differences in awareness and salience of health warning labels on smokeless tobacco between pre- and post-policy periods (Waves 1 and 2) among all pre-and post-policy completers (*N* = 5142) those that quit SLT by post-policy (*n* = 508). (DOC 40 kb)

## Abbreviations

GEE: Generalized estimated equations
HWL: Health warning label
SEAR: South East Asian Region
TCP: Tobacco control project
WHO FCTC: World health organization Framework Convention on Tobacco Control
