# Supplementary material for: An examination of the effectiveness of health warning labels on smokeless tobacco products in four states in India: findings from the TCP India cohort survey
Source: BMC Public Health. 2016 Dec 13;16:1246. doi: 10.1186/s12889-016-3899-7 (PMC5154141; doi:10.1186/s12889-016-3899-7)
Supplement: Additional file 1: Table S1. — Type of products used by SLT respondents by state among those who reported using only one product. (DOC 50 kb) [file 12889_2016_3899_MOESM1_ESM.doc]

Additional file 1: Table S1. Type of products used by SLT respondents by state among those who reported using only one product.

| **State** | | | | | | | | | | | | | | | | |
| --- | --- | --- | --- | --- | --- | --- | --- | --- | --- | --- | --- | --- | --- | --- | --- | --- |
|  | **Bihar (n = 1224)** | | | | **West Bengal (n = 673)** | | | | **Madhya Pradesh (n = 773)** | | | | **Maharashtra (n = 839)** | | | |
| **Smokeless  Product** | **N** | **%** | **(95%** | **CI)** | **N** | **%** | **(95%** | **CI)** | **N** | **%** | **(95%** | **CI)** | **N** | **%** | **(95%** | **CI)** |
| Mishri | 0 |  |  |  | 0 |  |  |  | 0 |  |  |  | 315 | **36.8** | 29.6 | 44.6 |
| Betel quid | 26 | **1.7** | 0.5 | 4.4 | 37 | **6.8** | 2.1 | 19.8 | 4 | **0.3** | 0.0 | 1.3 | 106 | **12.3** | 6.7 | 21.5 |
| Chewing tobacco | 2 | **0.1** | 0.0 | 0.4 | 4 | **0.8** | 0.1 | 2.5 | 310 | **41.0** | 32.5 | 50.2 | 206 | **26.9** | 19.5 | 35.8 |
| Gutka | 94 | **5.1** | 3.3 | 7.9 | 127 | **16.3** | 12.2 | 21.6 | 361 | **46.6** | 35.4 | 58.3 | 60 | **9.0** | 6.7 | 12.0 |
| Khaini | 408 | **44.7** | 34.6 | 55.3 | 152 | **26.2** | 19.4 | 34.4 | 8 | **1.3** | 0.5 | 2.7 | 9 | **1.4** | 0.6 | 2.6 |
| Zarda | 9 | **0.3** | 0.1 | 1.1 | 68 | **9.4** | 4.8 | 17.7 | 71 | **8.4** | 3.4 | 19.2 | 34 | **4.8** | 0.7 | 14.9 |
| Tobacco paste | 4 | **0.1** | 0.0 | 0.6 | 3 | **0.3** | 0.0 | 1.1 | 9 | **0.9** | 0.1 | 3.2 | 1 | **0.1** | 0.0 | 0.7 |
| Snuff | 0 |  |  |  | 21 | **2.7** | 0.9 | 6.1 | 8 | **1.1** | 0.0 | 5.3 | 87 | **6.4** | 1.6 | 22.9 |
| Lal dantmanjan | 637 | **44.8** | 36.2 | 53.7 | 1 | **0.1** | 0.0 | 0.7 | 0 |  |  |  | 10 | **0.9** | 0.3 | 2.3 |
| Dokta | 1 | **0.0** | 0.0 | 0.4 | 10 | **1.3** | 0.4 | 3.0 | 0 |  |  |  | 0 |  |  |  |
| Gudhaku | 2 | **0.1** | 0.0 | 0.4 | 163 | **22.0** | 14.0 | 32.7 | 0 |  |  |  | 0 |  |  |  |
| Gul | 41 | **3.0** | 0.9 | 7.4 | 53 | **6.0** | 2.9 | 12.0 | 2 | **0.3** | 0.0 | 1.7 | 4 | **0.4** | 0.1 | 1.2 |
| Other | 0 |  |  |  | 34 | **8.0** | 1.6 | 31.4 | 0 |  |  |  | 7 | **0.9** | 0.2 | 3.0 |

Frequencies are unweighted and all other data (estimates and CIs) are weighted
