# Supplementary material for: An examination of the effectiveness of health warning labels on smokeless tobacco products in four states in India: findings from the TCP India cohort survey
Source: BMC Public Health. 2016 Dec 13;16:1246. doi: 10.1186/s12889-016-3899-7 (PMC5154141; doi:10.1186/s12889-016-3899-7)
Supplement: Additional file 2: Table S2. — GEE analysis examining differences in awareness and salience of health warning labels on smokeless tobacco between pre- and post-policy periods (Waves 1 and 2) among all pre-and post-policy completers (N = 5142) those that quit SLT by post-policy (n = 508). (DOC 40 kb) [file 12889_2016_3899_MOESM2_ESM.doc]

Additional file 2: Table S2. GEE analysis examining differences in awareness and salience of health warning labels on smokeless tobacco between pre- and post-policy periods (Waves 1 and 2) among all pre-and post-policy completers (N=5,142)those that quit SLT by post-policy (n=508).

|  | **Wave** | | | **Wave** | | |  | | | |
| --- | --- | --- | --- | --- | --- | --- | --- | --- | --- | --- |
|  | **1** | | | **2** | | | **Difference between Waves** | | | |
| **Outcome** | **%** | **(95%** | **CI)** | **%** | **(95%** | **CI)** | **Diff** | **SE Diff** | **Test** | **p value** |
| **All pre-and post-policy completers (N=5,142)** |  |  |  |  |  |  |  |  |  |  |
|  |  |  |  |  |  |  |  |  |  |  |
| Aware that SLT packages contain HWLs (yes) | **73.2** | 67.5 | 78.2 | **74.3** | 68.7 | 79.3 | 1.1 | 2.9 | 0.4 | 0.693 |
|  |  |  |  |  |  |  |  |  |  |  |
| Noticed HWLs at least once in a while (yes) | **35.5** | 29.4 | 42.1 | **28.2** | 21.7 | 35.9 | -7.3 | 4.3 | -1.7 | 0.097 |
| **Respondents that quit SLT by post-policy** (N=508)* |  |  |  |  |  |  |  |  |  |  |
| Aware that SLT packages contain HWLs (yes) | **77.8** | 65.8 | 86.5 | **86.8** | 78.5 | 92.2 | 9.0 | 3.7 | 2.4 | 0.020 |
|  |  |  |  |  |  |  |  |  |  |  |
| Noticed HWLs at least once in a while (yes) | **47.2** | 32.5 | 62.3 | **29.9** | 18.2 | 45.0 | -17.3 | 9.9 | -1.7 | 0.088 |

Note: *****519 Pre-policy respondents reported quitting SLT by post-policy and 508 had complete data for analyses.
